# A Scoring System for Assessing the Risk of Malignant Partially Cystic Thyroid Nodules Based on Ultrasound Features

**DOI:** 10.3389/fonc.2021.731779

**Published:** 2021-10-06

**Authors:** Yuwei Xin, Feifei Liu, Yan Shi, Xiaohui Yan, Liping Liu, Jiaan Zhu

**Affiliations:** ^1^ Department of Ultrasound, First Hospital of Shanxi Medical University, Taiyuan, China; ^2^ Department of Ultrasound, Peking University People’s Hospital, Beijing, China; ^3^ Department of Ultrasound, Binzhou Medical University Hospital, Binzhou, China

**Keywords:** partially cystic thyroid nodules, ultrasound features, scoring system, prediction model, malignant risk

## Abstract

**Objective:**

To assess the ultrasound (US) features of partially cystic thyroid nodules (PCTNs) and to establish a scoring system to further improve the diagnostic accuracy.

**Methods:**

A total of 262 consecutive nodules from September 2017 to March 2020 were included in a primary cohort to construct a scoring system. Moreover, 83 consecutive nodules were enrolled as an validation cohort from May 2018 to August 2020. All nodules were determined to be benign or malignant according to the pathological results after surgery or ultrasound-guided fine-needle aspiration (US-FNA). The US images and demographic characteristics of the patients were analyzed. The ultrasound features of PCTNs were extracted from primary cohort by two experienced radiologists. The features extracted were used to develop a scoring system using logistic regression analysis. Receiver operating characteristic (ROC) curves were applied to evaluate the diagnostic efficacy of the scoring system in both the primary cohort and validation cohort. In addition, the radiologists evaluated the benign and malignant PCTNs of the validation cohort according to the ACR TI-RADS guidelines and clinical experience, and the accuracy of their diagnosis were compared with that of the scoring system.

**Results:**

Based on the eight features of PCTNs, the scoring system showed good differentiation and reproducibility in both cohorts. The scoring system was based on eight features of PCTNs and showed good performance. The area under the curve (AUC) was 0.876 (95% CI, 0.830 - 0.913) in the primary cohort and 0.829(95% CI, 0.730 - 0.903) in the validation cohort. The optimal cutoff value of the scoring system for the diagnosis of malignant PCTNs was 4 points, with a good sensitivity of 71.05% and specificity of 87.63%. The scoring system (AUC=0.829) was superior to radiologists (AUC= 0.736) in diagnosing PCTNs and is a promising method for clinical application.

**Conclusions:**

The scoring system described herein is a convenient and clinically valuable method that can diagnose PCTNs with relatively high accuracy. The use of this method to diagnose PCTNs, which have been previously underestimated, will allow PCTNs to receive reasonable attention, and assist radiologist to confidently diagnose the benignity or malignancy.

## Introduction

With the application of high-resolution ultrasound, thyroid nodules are found in 19%–67% of ultrasound exams (1). A portion of them (15%-53.8%) are mixed echoic nodules, which have both cystic and solid components (2, 3). Partially cystic thyroid nodules (PCTNs) are considered to result from cystic degeneration due to hemorrhage, ischemia, necrosis and liquefaction. Nodules with cystic degeneration in the thyroid gland, such as nodular goiters and thyroid adenoma, are mostly benign lesions. Previous studies have shown that the proportion of cystic components in nodules is inversely proportional to their malignant potential (3–5).

However, 13%-26% of thyroid carcinomas may have cystic degeneration (6). For PCTNs, the percentage of malignancy varies from approximately 2% to 18% (1, 2, 7). PCTNs are often underestimated. Currently, the ultrasound-guided fine needle aspiration (US-FNA) of thyroid nodules is recognized as the most reliable method for preoperative differential diagnosis. This method, however, may be limited by several factors, such as its invasiveness, insufficient sampling and/or indeterminate cytology, and operator dependence (8–10). Moreover, the presence of cystic components in PCTNs may further reduce the diagnostic accuracy of FNA (11). There are few studies on the US features related to malignant PCTN, and these features differ from those of solid nodules (12–15). Therefore, the purpose of this study was to determine the ultrasound features that predict the malignancy of PCTNs and to analyze the correlations to construct a scoring system and present it as a forest plot. Such a scoring system is expected to improve the preoperative diagnostic efficacy and to facilitate patient management in clinical practice.

## Materials and Methods

### Patients

This study was retrospective and was approved by the Ethics Review Committee of First Hospital of Shanxi Medical University; the requirement for informed consent was waived. The inclusion and exclusion criteria for the study were as follows.

Inclusion criteria: (I) the composition of the nodule was mixed echo, (II) the patients underwent high-resolution ultrasound examination within one week before clinical intervention, (III) the pathology results of the nodules were confirmed after surgery or US-FNA, (IV) the patients underwent an initial surgery, and (V) the patients were not treated with any preoperative radiotherapy, chemotherapy and endocrine therapies.

The exclusion criteria were as follows: (I) family history of thyroid carcinoma, (II) head and neck exposure to high doses of radiation, (III) combined nonthyroid metastatic tumors in other parts, (IV) other endocrine system diseases and other metabolic diseases, and (V) incomplete clinical and pathological information.

Finally, a total of 262 patients with PTCNs were enrolled consecutively as a primary cohort to construct the scoring system and conduct internal validation from September 2017 to March 2020. The mean age of the patients was 49.4 ± 12.5 years (range: 18–84 years), and 63 males and 199 females were included. Next, to validate the efficacy of the PCTN scoring system, another 83 PTCN patients were included at a 3:1 ratio as the validation cohort from May 2018 to August 2020. The mean age of these patients was 46.8 ± 13.0 years (range: 21–75 years), and19 males and 64 females were included. The same inclusion and exclusion criteria were used for the validation cohort.

The clinical data, US images and histopathological/cytopathology results were obtained from patients who underwent surgery or FNA.

### Ultrasonography Evaluation

All patients were examined by preoperative thyroid ultrasonography in our department. The high-resolution US images were acquired by two commercial machines, iU22 (Philip, Amsterdam, Holland) equipped with a 5-12 MHz and Logic E9 (GE medical systems, Milwaukee, WI, USA) equipped with a 6-15 MHz linear-phased array transducer. The parameters were adjusted to those that were the best and consistent among patients, and the focus was set at the level of the target nodules. The grayscale and color Doppler ultrasonic images of both longitudinal and cross sections were acquired continuously, and the images of thyroid lesions were then stored in the DICOM format for further analysis. Two radiologists with more than 10 years of experience performing thyroid ultrasound examinations were responsible for the image acquisition and ultrasound feature assessment and were blinded to the patients’ medical histories. The reproducibility of the US feature evaluation was assessed based on the degree of inter- and intraobserver agreement. The US features were evaluated by radiologist 1 in the primary cohort and re-evaluated a week later to assess the intraoperator agreement. Radiologist 2 performed the same examination in the primary cohort and compared the findings with those of radiologist 1 to evaluate the interoperator agreement. The interval between the two radiologists was within 30 minutes.

**Figure 1:**
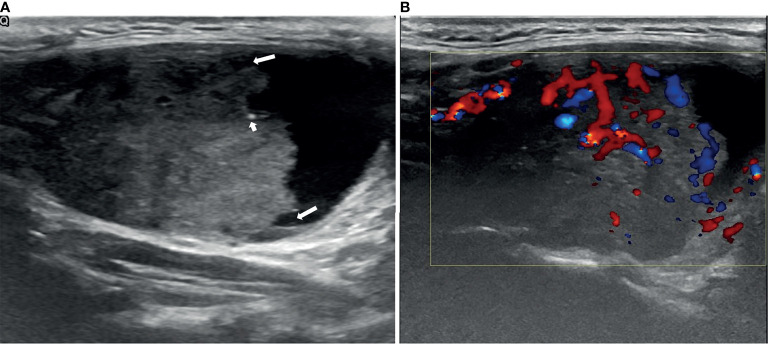
An example of a PCTN with an eccentric solid position with an acute angle (long arrows) in a 38-year-old woman. Longitudinal ultrasound (US) image of a 5.06×3.07cm nodule with internal solid composition (accounted for approximately 64% of the total PCTN volume.) showing ill-defined margin and a little micro-calcification (short arrows) **(A)**. CDFI showing internal and peripheral flow inside the solid component **(B)**. Papillary thyroid carcinoma was diagnosed by fine needle aspiration (FNA) and confirmed by surgical histopathology.

**Figure 2:**
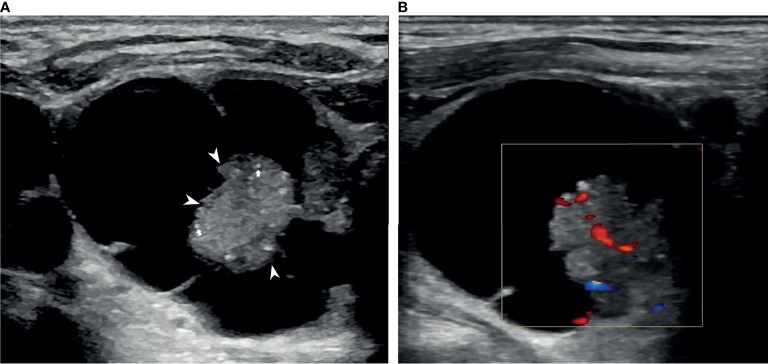
An example of a PCTN with a concentric solid position in a 76-year-old man. Transverse ultrasound (US) image of a 3.43×3.10 cm nodule with internal solid composition (size of 1.49×1.44 cm) showing non-smooth free-margin (arrowheads) and multiple micro-calcifications (arrows) **(A)**. CDFI showing internal flow inside the solid component **(B)**. Papillary thyroid carcinoma was diagnosed by fine needle aspiration (FNA) and confirmed by surgical histopathology.

The nodules were evaluated according to their 1) composition (solid portion <50% *vs* solid portion ≥50%), 2) position of the solid portion (concentric *vs.* eccentric blunt angle *vs*. eccentric acute angle), 3) shape (ovoid-to-round *vs*. taller-than-wide), 4) margins (smooth *vs*. spiculated/microlobulated *vs*. ill-defined), 5) echogenicity of the solid portion (marked hypoechoic *vs*. hypoechoic *vs*. isoechoic/hyperechoic), 6) free margin (smooth *vs*. nonsmooth, which was defined as the interface between the cystic and solid components, **Figure 1**), 7) calcification (microcalcifications *vs*. rim calcifications *vs*. macrocalcifications *vs*. no calcification or comet tail, **Figure 2**), and 8) vascularity (intranodular *vs*. peripheral *vs*. avascular).

US-FNA was performed by the same radiologist who performed the US examination using a 21-gauge needle. When the cystic portion was larger than 50% of the nodule, FNA for the solid portion was performed after the fluid was aspirated. Afterwards, the interpretation of FNA was based on the Bethesda System for Reporting Thyroid Cytopathology (16). The cytological results of inadequate (Bethesda Category I) and indeterminate (Bethesda Category III) without surgical outcome were excluded in this study. PCTNs falling into the Bethesda Category II with a minimum follow-up of 6 months were considered benign nodules. The final diagnosis of all malignant PCTNs were verified by histopathology after surgery.

### Statistical Analysis

Statistical analysis was performed using SPSS version 22.0 (SPSS Inc., Chicago, IL, USA). Continuous variables are expressed as the mean ± SD, and categorical variables are expressed as percentages (%). The continuous variables were compared using Student’s t-test and the F test. Categorical variables were compared using Pearson’s chi-squared test or Fisher’s exact test. A *p* value <0.05 was considered statistically significant. The *k* value was used to evaluate the degrees of intraobserver and interobserver agreement. We used a scoring system to simulate logistic regression analyses in the primary cohort to differentiate malignant and benign PCTNs. All US features were encapsulated in the logistic regression model and are presented as forest plots. The coefficients for each ultrasound feature can be obtained, which represents its weight in the scoring system. The coefficients were standardized using rounding to obtain the score of each feature to construct the scoring system. The goodness-of-fit of the logistic regression models was evaluated using the Hosmer-Lemeshow test. Receiver operator characteristic (ROC) analysis was used to illustrate the diagnostic thresholds and assess the performance of the scoring system in both the primary cohort and validation cohort. The sensitivities and specificities as well as the accuracy of the diagnosis were calculated. The AUC values of the different diagnostic evaluation methods were compared using the DeLong method.

A power calculation was performed using the PASS 15 (Power Analysis and Sample Size Software, 2017, NCSS, LLC. Kaysville, Utah, USA, ncss.com/software/pass) “Tests for One ROC Curve” function.

## Results

### Clinical Characteristics and Ultrasonic Features

The baseline clinical characteristics and ultrasonic features of the 262 patients in the primary cohort and 83 patients in the validation cohort are summarized in **Table 1**. Of the 345 nodules, 251 (72.8%) were finally diagnosed as benign, while 94 (27.2%) were diagnosed as malignant. Among the 262 benign lesions, 56 (45 nodular hyperplasias, 8 follicular adenomas, and 3 Hashimoto’s nodules) were confirmed by surgery due to suspicion of carcinoma by FNA or compression symptoms caused by large nodules. The remaining benign nodules were confirmed by FNA and were followed up for at least 6 months. All malignant nodules, which included 79 papillary carcinomas, 9 follicular carcinomas, and 6 anaplastic carcinomas, were surgically resected.

**Table 1:** Baseline characteristics in the primary cohort and validation cohort.

Characteristic	Primary cohort (N = 262)			Validation cohort (N = 83)		
	Benign nodules	Malignant nodules	P	Benign nodules	Malignant nodules	P
	(N = 186)	(N = 76)		(N = 65)	(N = 18)	
Age, mean ± SD, years	48.6 ± 13.0	50.1 ± 12.0	0.173	46.9 ± 13.4	46.7 ± 12.6	0.948
Sex, No.%			0.943			1.000
Male	44 (23.7%)	19 (25.0%)		15 (23.1%)	4 (22.2%)	
Female	142 (76.3%)	57 (75.0%)		50 (76.9%)	14 (77.8%)	
Size, cm	3.35 ± 1.21	3.45 ± 1.07	0.999	3.15 ± 1.16	3.21 ± 0.98	0.835
Composition, No.%			0.001			0.276
Solid portion <50%	108 (58.1%)	26 (34.2%)		28 (43.1%)	11 (61.1%)	
Solid portion ≥50%	78 (41.9%)	50 (65.8%)		37 (56.9%)	7 (38.9%)	
Position, No.%			<0.001			0.001
Concentric	105 (56.5%)	27 (35.5%)		35 (53.8%)	4 (22.2%)	
Eccentric blunt angle	67 (36.0%)	17 (22.4%)		24 (36.9%)	5 (27.8%)	
Eccentric acute angle	14 (7.53%)	32 (42.1%)		6 (9.23%)	9 (50.0%)	
Shape, No.%			0.003			0.331
A/T<1	117 (62.9%)	32 (42.1%)		36 (55.4%)	7 (38.9%)	
A/T≥1	69 (37.1%)	44 (57.9%)		29 (44.6%)	11 (61.1%)	
Margin, No.%			<0.011			0.437
Smooth	75 (40.3%)	18 (23.7%)		25 (38.5%)	4 (22.2%)	
Ill-defined	67 (36.0%)	28 (36.8%)		22 (33.8%)	8 (44.4%)	
Spiculated/microlobulated	44 (23.7%)	30 (39.5%)		18 (27.7%)	6 (33.3%)	
Echogenicity, No.%			0.004			0.106
Hyperechoic/isoechoic	12 (6.45%)	1 (1.32%)		2 (3.08%)	1 (5.56%)	
Hypoechoic	116 (62.4%)	36 (47.4%)		45(69.2%)	8 (44.4%)	
Marked hypoechoic	58 (31.2%)	39 (51.3%)		18 (27.7%)	9 (50.0%)	
Free - margin, No.%			<0.001			0.392
Smooth	114 (61.3%)	24 (31.6%)		35 (53.8%)	7 (38.9%)	
Non-smooth	72 (38.7%)	52 (68.4%)		30 (46.2%)	11 (61.1%)	
Calcification, No.%			<0.001			0.016
No calcification	110 (59.1%)	25 (32.9%)		42 (64.6%)	7 (38.9%)	
Macrocalcification	36 (19.4%)	9 (11.8%)		8 (12.3%)	3 (16.7%)	
Rim calcification	35 (18.8%)	9 (11.8%)		11 (16.9%)	2 (11.1%)	
Microcalcification	5 (2.69%)	33 (43.4%)		4 (6.15%)	6 (33.3%)	
Color Doppler, No.%			0.181			0.128
Avascular	78 (41.9%)	26 (34.2%)		26 (40.0%)	3 (16.7%)	
Peripheral blood flow	45 (24.2%)	15 (19.7%)		15 (23.1%)	4 (22.2%)	
Internal flow	63 (33.9%)	35 (46.1%)		24 (36.9%)	11 (61.1%)	

The sexes, ages and nodule sizes (the longest diameter) were not significantly different between patients with malignant and benign PCTNs.

### System for Scoring the Ultrasonic Features in the Primary Cohort

The intraoperator *k* value of radiologist 1 in the two examinations ranged from 0.86 to 0.96. The interoperator *k* value of examinations by radiologist 1 and radiologist 2 ranged from 0.82 to 0.92, suggesting that the extraction of US features was stable and repeatable. After the inter- and intraoperator repeatability were verified, all results were based on the features extracted by radiologist 1.

In the primary cohort, we preset the power = 0.90, α = 0.05, R^2^ = 0.36, and the sample size meeting the requirements was determined to be 280. In the validation cohort, the AUCs were 0.81 (95% confidence interval (CI): 0.709-0.883) and 0.70 (95% CI: 0.591-0.791); we thus preset AUC1 = 0.80-0.90, the α = 0.05, and the false positive rate limits = 0.01-0.20. The sample size was 92, and the validation cohort was of sufficient size to evaluate the AUC estimated from the primary cohort (**PASS output File 1** and **File 2** in **Supplementary Material**).

**Figure 3:**
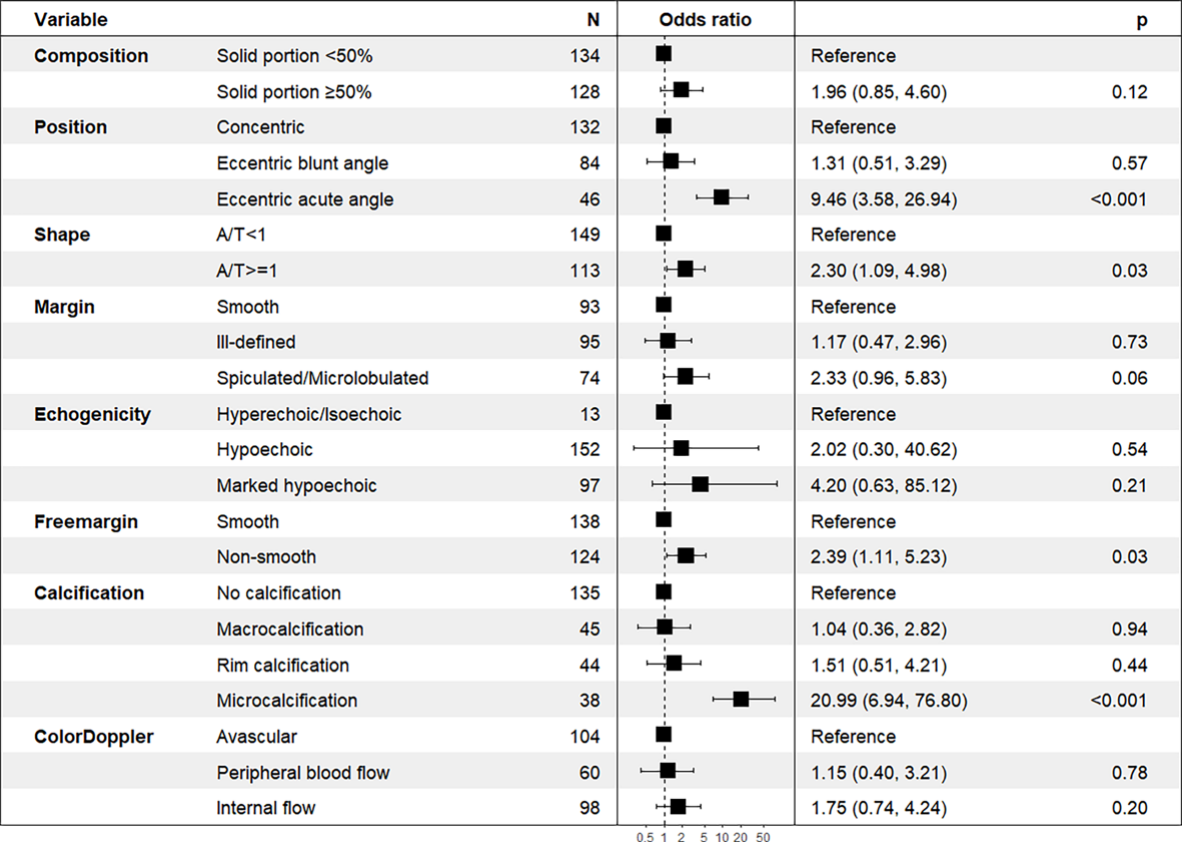
A forest plot of the logistic regression analysis results.

**Figure 4:**
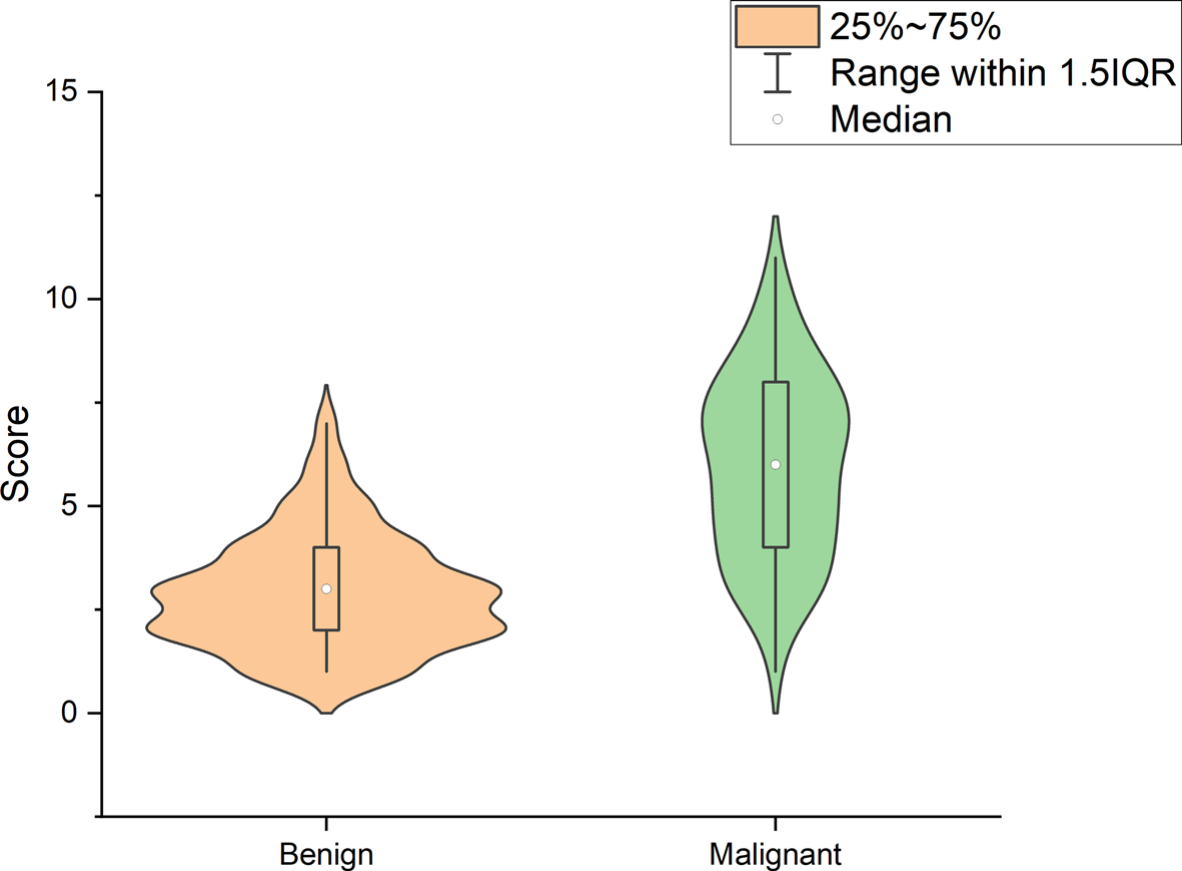
The distributions of all PCTNs in the primary cohort in the scoring system shows as Violin plot.

**Figure 5:**
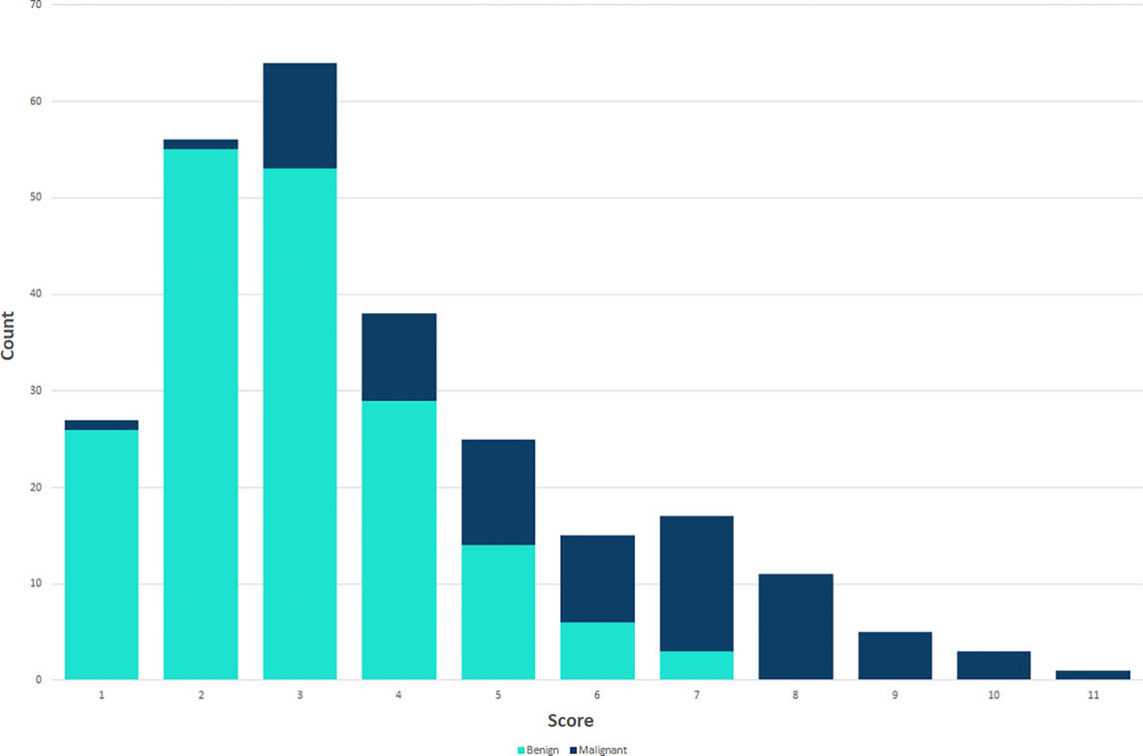
The distributions of all PCTNs in the primary cohort in the scoring system shows as bar plot.

**Figure 6:**
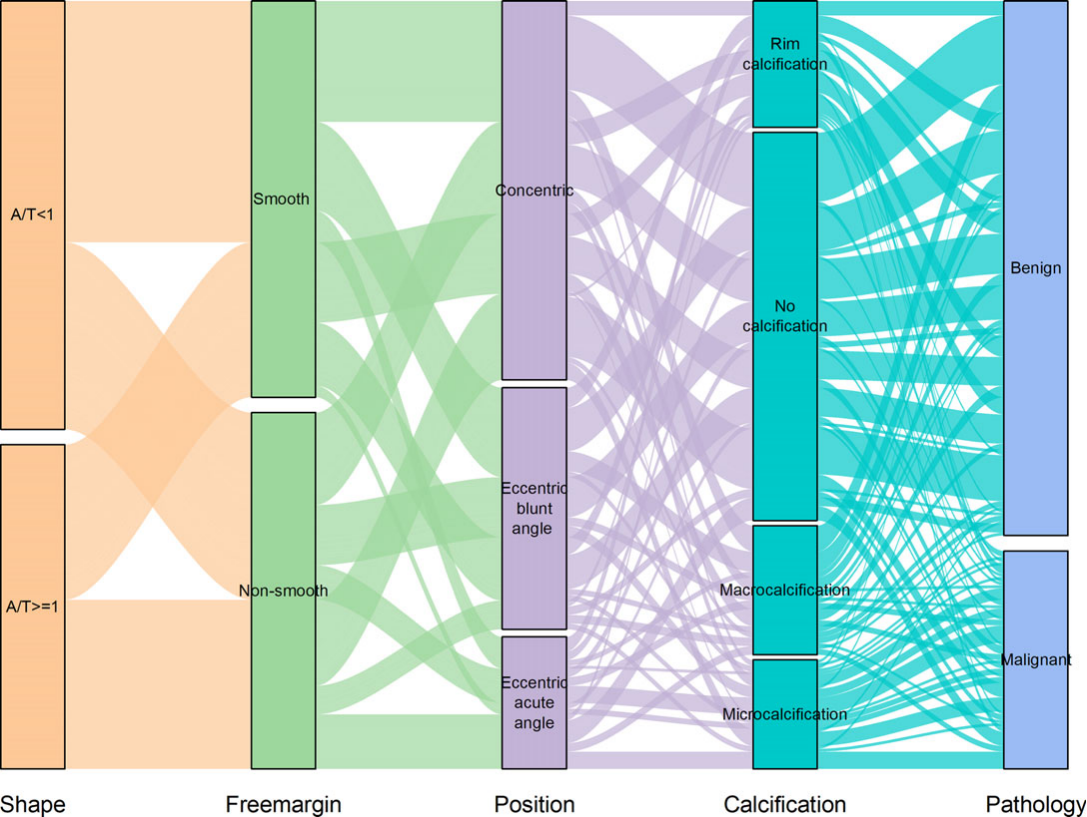
The distributions of all PCTNs in the primary cohort in the ultrasound features shows as Sankey plot.

Using logistic regression analysis of the primary cohort, eight US features of PCTNs were finally incorporated into the scoring system. The logistic regression analysis results were presented as a forest plot (**Figure 3**). To promote facilitation in clinical practice, we used a standardized coefficient to convert the logistic regression model into a scoring system to distinguish benign and malignant nodules, which meant that we rounded off the coefficients of the logistic regression model. The Hosmer-Lemeshow test of the logistics regression model was shown in **Supplementary Figure 1**. The scoring system was presented in **Table 2**, and the score ranged from 0-11 points. The distributions of benign and malignant nodules in the scoring system and ultrasound features were shown as the Violin plot and bar plot (**Figures 4**, **5**) and Sankey plot (**Figure 6**), respectively.

**Table 2:** Scoring table of the scoring system.

US features		Score
Composition	Solid portion <50%	0
	Solid portion >=50%	1
Position	Concentric	0
	Eccentric blunt angle	0
	Eccentric acute angle	2
Shape	A/T<1	0
	A/T>=1	1
Margin	Smooth	0
	Ill-defined	0
	Spiculated/Microlobulated	1
Echogenicity	Hyperechoic/Isoechoic	0
	Hypoechoic	1
	Marked hypoechoic	1
Free - margin	Smooth	0
	Non-smooth	1
Calcification	No calcification	0
	Macrocalcification	0
	Rim calcification	0
	Microcalcification	3
Color Doppler	Avascular	0
	Peripheral blood flow	0
	Internal flow	1

**Figure 7:**
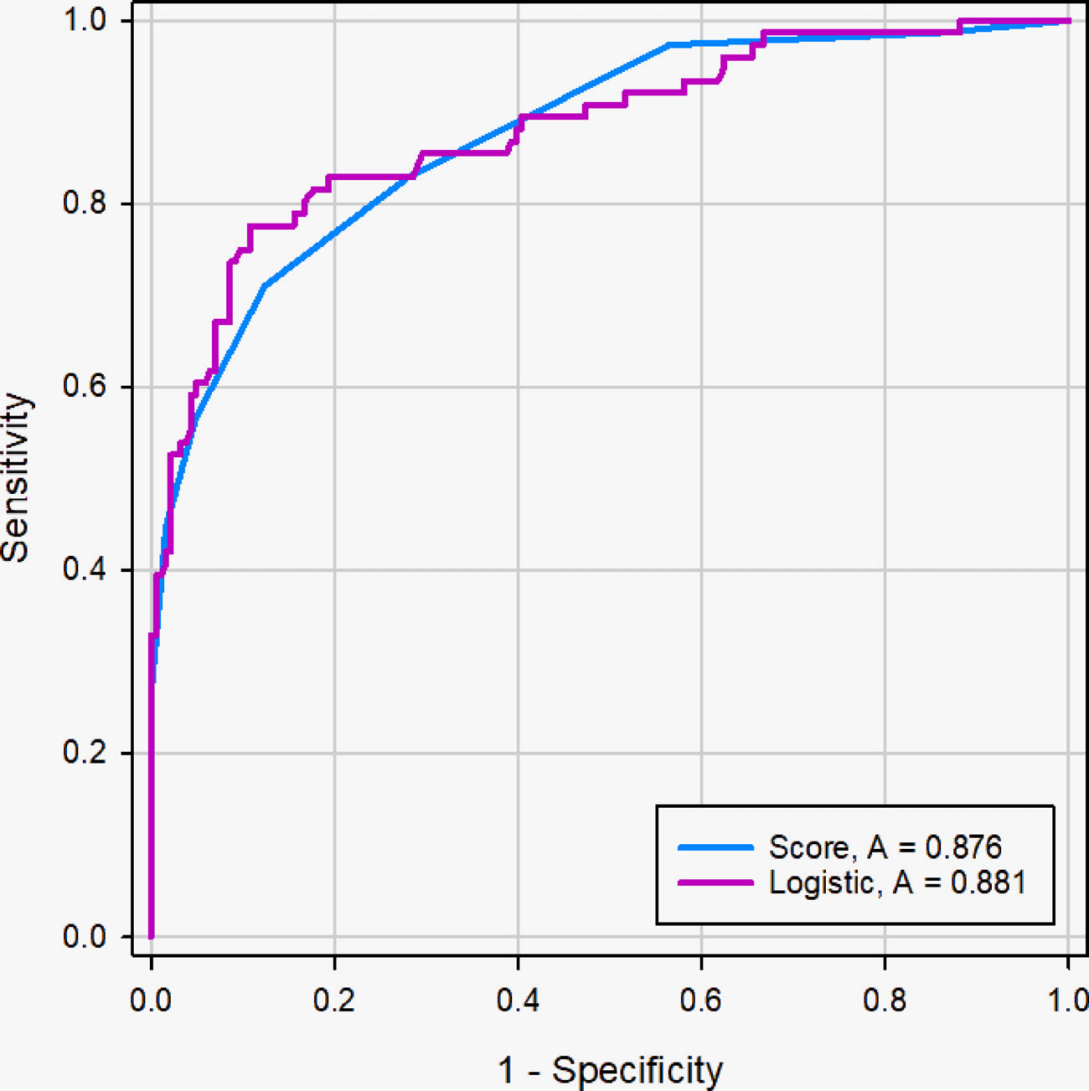
ROC curve of logistic regression models and scoring system in the primary cohort. The difference of AUC was not significant between two models (0. 881 *vs.* 0. 876), and the DeLong et al. indicated that there was not significant difference in diagnostic performance between two models (*p* =0.66).

The ROC curve showed good discriminating power, with AUCs of 0.881 [95% CI, 0.836 - 0.918] in the logistic regression model and 0.876 (95% CI, 0.830 - 0.913) in the scoring system (**Figure 7**). The difference was not significant as determined by the DeLong et al. method (*p* = 0.66). Therefore, it was reasonable to assume that the scoring system actually simulated the logistic regression model. The diagnostic performance of the scoring system in the primary cohort was shown in **Table 3**. The sensitivity and specificity for all scores of primary cohort and validation cohort were presented in **Supplementary Table 1**. The optimal cutoff value for the diagnosis of malignant PCTNs was 4 points, with a good specificity of 87.63% and a sensitivity of 71.05%. Furthermore, to show the diagnostic ability of the scoring system, we created a confusion matrix in the primary cohort and validation cohort, as shown in **Supplementary Figures 2A, B**.

**Table 3:** Diagnostic performance of the scoring system in primary and validation cohort.

Diagnostic performance	Primary cohort	Validation cohort
AUC (95% CI)	0.876 (0.830 - 0.913)	0.829 (0.730 - 0.903)
Specificity (%) (95% CI)	87.63 (82.0 - 92.0)	72.31 (59.8 - 82.7)
Sensitivity (%) (95% CI)	71.05 (59.5 - 80.9)	77.78 (52.4 - 93.6)
PPV (%) (95% CI)	70.1 (60.9 - 77.9)	43.7 (32.8 - 55.3)
NPV (%) (95% CI)	88.1 (83.8 - 91.4)	92.2 (83.0 - 96.6)
Accuracy	0.83	0.74

The optimal cutoff value is set to 4 points both in the primary cohort and validation cohort. PPV, Positive predictive value; NPV, Negative predictive value.

### Validation of the Scoring System with the Validation Cohort

The AUC of the scoring system was 0.829 (95% CI, 0.730 - 0.903) in the validation cohort. The diagnostic performance of the scoring system in the validation cohort was shown in **Table 3**. The optimal cutoff value was the same as that in the primary cohort.

### Comparison of the Diagnostic Accuracies of the Scoring System and Radiologists

Radiologist1 evaluated the PCTNs of the validation cohort based on the American College of Radiology guidelines (2017 version) (17) and clinical experience and classified the nodules as either malignant or benign. We then compared the accuracies of the radiologists and the scoring system to diagnose PTCNs.

**Figure 8:**
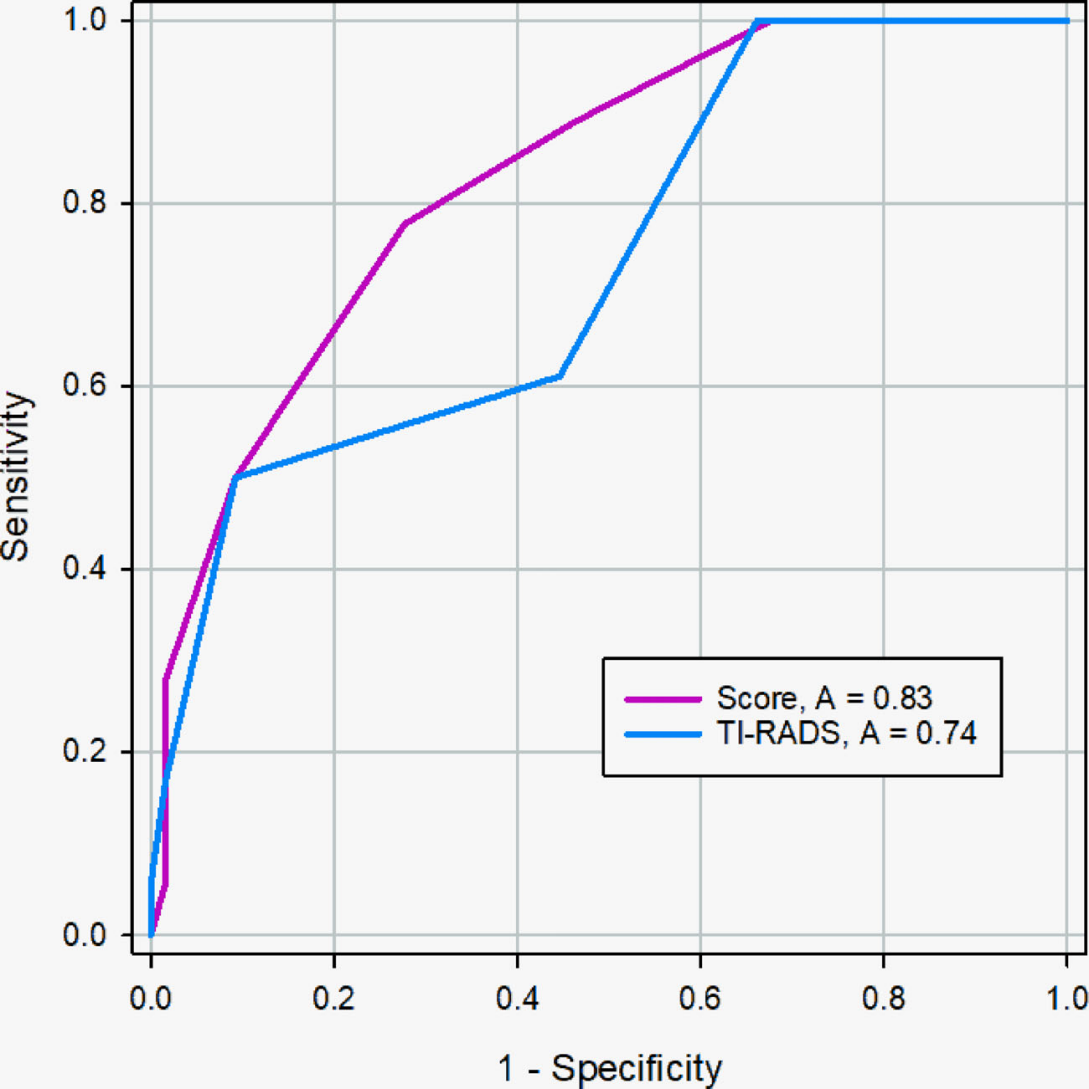
ROC curve of the scoring system and radiologists in the validation cohort. The AUC was significantly different between two methods (0.83 *vs.* 0.74), and the DeLong et al. indicated that there was a significant difference in diagnostic performance between two methods (*p* = 0.041).

The AUC of the scoring system was 0.829 (95% CI, 0.730 - 0.903), while that of the radiologists was 0.736 (95% CI, 0.628 - 0.827) in the validation cohort (**Figure 8**). The difference was significant as determined by the DeLong method (*p* = 0.041).

### Nodule Size and Its Influence on the Performance of the Scoring System

**Figure 9:**
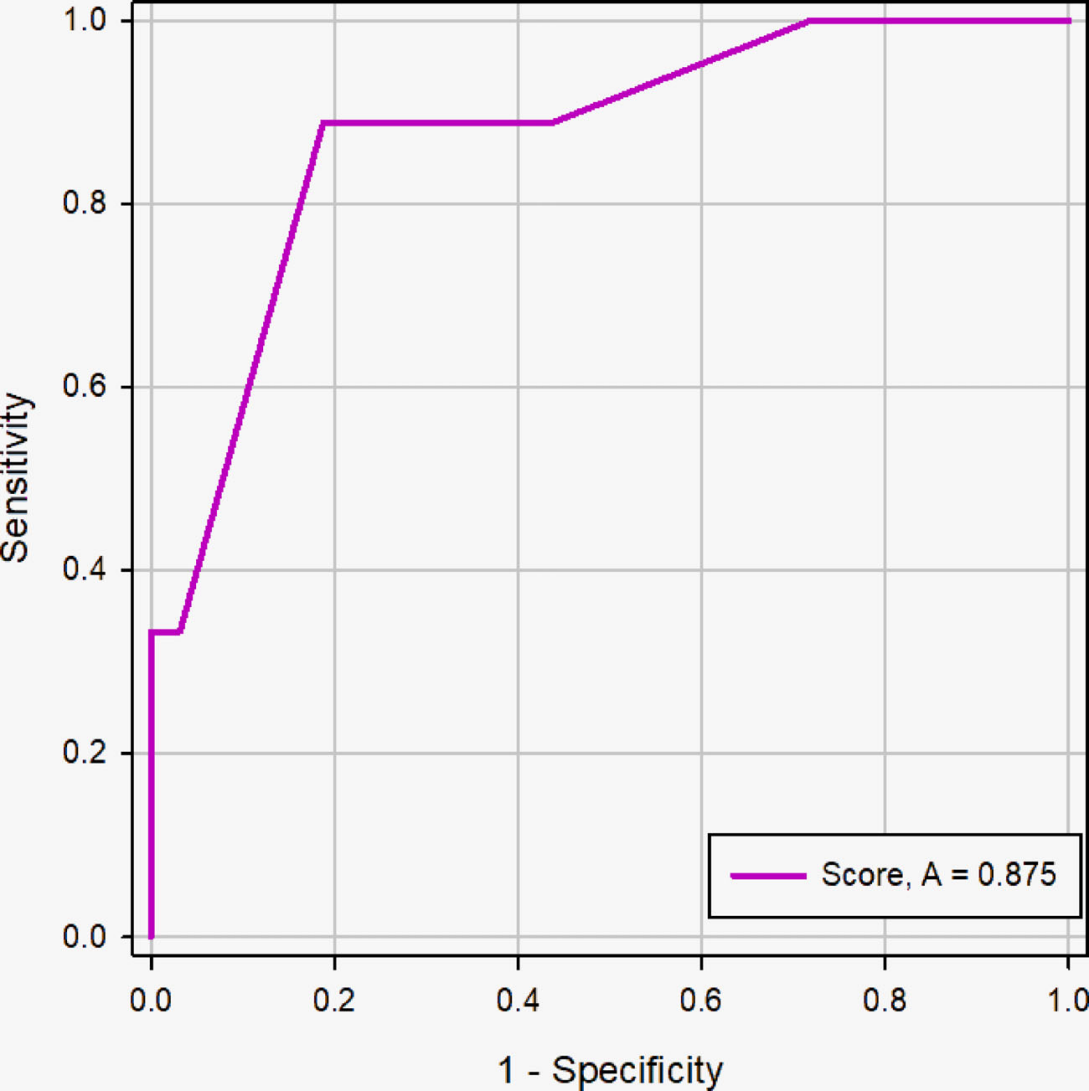
ROC curve of the scoring system in the nodule size <2cm group. The AUC was 0.875 (95% CI: 0.734 - 0.957, *p <*0.0001).

**Figure 10:**
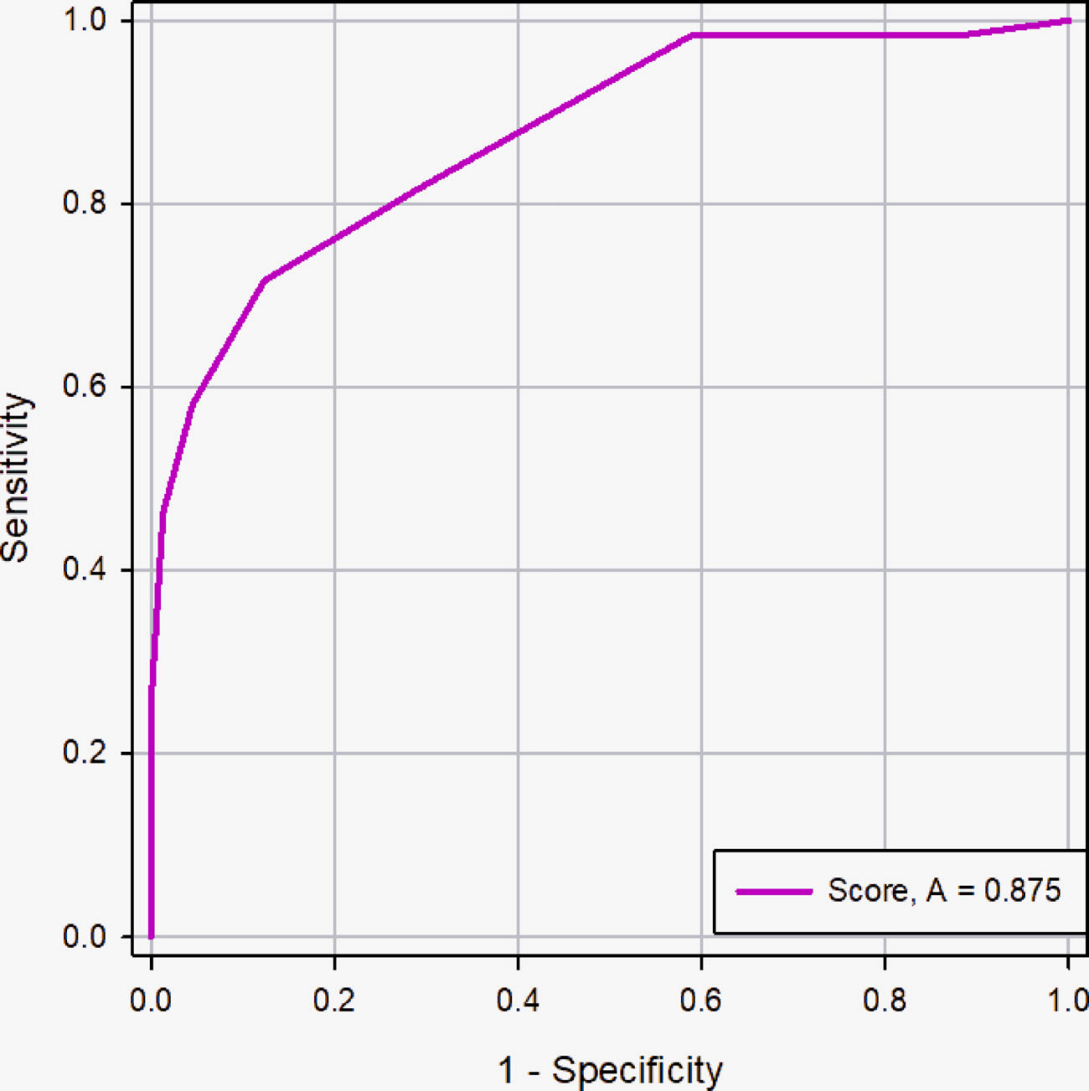
ROC curve of the scoring system in the nodule size ≥2cm group. The AUC was 0.875 (95% CI: 0.824 - 0.915, *p <*0.0001).

In this study, 262 patients in the primary cohort were divided into <2cm group (n=41) and ≥2cm group (n=221) based on the longest diameter of PCTNs and to evaluate the diagnostic efficacy of scoring system, further determining the influence of nodule size on scoring system. The AUC of the scoring system was for the prospective sonographic diagnosis of PCTNs of<2 cm and ≥ 2 cm were 0.875 (95% CI, 0.734 - 0.957) and 0.875 (95% CI, 0.824 -0.915), respectively (**Figures 9** and **10**). The diagnostic performance of the two groups was shown in **Table 4**. This result indicates high diagnostic value of the scoring system both in <2cm group and ≥2cm group.

**Table 4:** Diagnostic performance of the scoring system in nodule size <2cm and ≥2cm group.

Diagnostic performance	nodule size＜2cm	nodule size≥2cm
AUC (95% CI)	0.875 (0.734 - 0.957)	0.875 (0.824 - 0.915)
Specificity (%) (95% CI)	87.50 (71.0 - 96.5)	87.66 (81.4 - 92.4)
Sensitivity (%) (95% CI)	66.67 (29.9 - 92.5)	71.64 (59.3 - 82.0)
PPV (%) (95% CI)	60.0 (35.0 - 80.7)	71.6 (61.8 - 79.8)
NPV (%) (95% CI)	90.3 (78.6 - 96.0)	87.7 (82.9 - 91.3)
Accuracy	0.829	0.828

The optimal cutoff value is set to 4 points both in nodule size＜2cm and ≥2cm group. PPV, Positive predictive value; NPV, Negative predictive value.

## Discussion

As a subtype of thyroid nodules, PCTNs are fairly common and can be differentiated easily according to conventional US features. Most PCTNs are regarded as benign, as degenerative processes arise in underlying benign lesions, such as nodular goiters and adenomatous nodules (18), and true epithelial cysts are rare. However, previous studies have revealed cystic changes in many malignant thyroid nodules, and the incidence of PCTN malignancy is up to 18% (1). PCTNs are easy to misdiagnose, especially for less experienced radiologists, due to their typical US features being different from those of solid nodules. The clinical management of PCTNs is still a challenge.

Ultrasound plays an essential role in evaluating the status of PCTNs because of its economical, noninvasive, and convenient features. Many studies have summarized the malignant features of solid thyroid nodules, such as their taller-than-wide shape, hypoechogenicity, and microcalcifications, while no clear diagnostic criteria have been established for malignant PCTNs. The sizes, compression symptoms, rates of recurrence after repeated aspirations, and local invasion of PCTNs are considered to be indicators of surgery (3, 19, 20). However, no general clinical features completely distinguish benign PCTNs from malignant PCTNs (3, 20, 21). Among 119 patients with PCTNs who underwent thyroidectomy after FNA, Bellantone (3) reported that 21 were malignant, yielding a 17.6% malignancy rate, and he therefore recommended FNA for all PCTNs; however, the malignancy rate among PCTNs may have been overestimated due to selection bias for the enrolled patients. Nevertheless, the presence of cystic components in a thyroid nodule may reduce the diagnostic efficiency of FNA. In this case, US-FNA is no longer recommended as a routine diagnostic method for PCTNs (22). Frates MC et al. (2) and LI W et al. (11) found that US features can help prioritize PCTNs for FNA. The main purpose of this study was to elucidate the US features that can predict PCTN malignancies and to construct a scoring system based on these US features to guide clinical practice.

Many studies have revealed the US features of malignant solid thyroid nodules, mainly including their echogenicity, margin, shape, microcalcification, and vascularity, which were all included in this study. In addition, PCTNs have unique nodule compositions, solid portion positions and free margins, and these characteristics were therefore also included in this study. The eccentric configuration of PCTNs was reported to indicate malignancy in a previous study (19); however, the sensitivity (44.4%) and positive predictive value (13.1%) were low. We further subdivided the eccentric configuration based on the angle to the adjacent cyst wall and found that only an acute angle was associated with malignancy (p <0.001), which was confirmed in the reports (12, 23). This phenomenon may be attributed to malignant PCTNs being more likely to protrude from the cystic wall, while diffuse lesions are circumferentially located within the cystic wall, forming a concentric configuration (24). Significantly, some researchers (13, 25) reported that when patients were regrouped into the ≥50% solid portion and <50% solid portion groups, an eccentric position of the solid component was a significantly malignant feature in only the solid portion ≥50% group. In our study, however, we did not find that a solid portion ≥50% was significantly associated with malignancy. This indicates that we should pay special attention to nodules that have both a solid portion ≥50% and an eccentric configuration.

A taller-than-wide shape was revealed to be an independent predictor of PCTN malignancy in our study. Previous studies found that the sensitivity and specificity of a taller-than-wide shape for the diagnosis of malignancy were 43.33% and 91.36%, respectively (26, 27). In contrast, some studies (12, 28) reported no significant correlation between a taller-than-wide shape and the risk of PCTN malignancy, which they suggested was potentially attributed to the inter- and intraobserver variability in taller-than-wide shapes.

In our study, we demonstrated that nonsmooth free margins were significantly more common in malignant thyroid nodules than in benign nodules. This may be illustrated by the histological tendencies of malignant PCTNs, including their uneven growth in an infiltrative manner and absence of pseudocapsule formation (14). Regarding the margins of entire nodules, no significant differences were found between benign and malignant PCTNs. Because large amounts of cystic fluid from benign lesions can be absorbed in a short time, the margin of the entire nodule is either smooth or ill-defined. Moreover, some malignant PCTNs can have smooth margins, especially those that are less than 10 mm in diameter.

It is well known that the presence of microcalcifications is highly specific for papillary thyroid carcinoma (14, 19). In this study, we defined microcalcifications as those less than 1 mm in size on US images; while this definition may not represent the actual pathological condition, all calcifications were confirmed by pathology. In this way, we excluded punctuate hyperechoic foci with comet tails, which may be another factor underlying misdiagnosis. Pathologically, nodule microcalcifications are correlated with psammoma bodies, which result from the degeneration of tumor cells and collagen produced by tumor cells (29).

Color Doppler ultrasound is a diagnostic tool for predicting thyroid carcinoma based on the hypothesis that peripheral or intranodular flow is caused by the angiogenesis-dependent growth of malignant tumors, while degenerating cystic lesions mainly present as the absence of internal flow signals or a small amount of peripheral flow. In this study, no significant differences were found among nodules with three different vascular patterns, which was possibly caused by rare blood flow signals detected in nodules during the period of stagnant growth (27).

To our knowledge, the risk of malignancy increases as more suspicious US features are detected in nodules (19). In our study, a logistic regression model was developed to assess the malignancy of PCTNs based on multiple ultrasonographic features. To make the model simple and convenient for clinical application, we proposed and validated a scoring system to predict the malignancy of PCTNs, which was based on the logistic regression model, achieving accuracies of 0.876 in the primary cohort and 0.829 in the validation cohort. A series of different TI-RADS guidelines have been proposed to evaluate solid thyroid nodules, although ACR TI-RADS has indeed taken the PCTN into consideration, but the ultrasound features in this guideline are mainly referred to solid thyroid nodules and the solid component of PCTN. This guideline has not fully considered the ultrasound features specific and important to PCTNs, such as free-margin, position of solid portion, and composition. Therefore, the strength of this scoring system was that it used eight common PCTN US features to construct the model. The scoring system displayed a remarkable ability to predict the malignancy of PCTNs (AUC=0.876), yielding a sensitivity of 71.05% and a specificity of 87.63%. These results indicate the potential value of our model for decreasing the rate of misdiagnosis and the use of US-FNA of PCTNs prior to surgery. Therefore, clinicians can now efficiently manage PCTN patients.

In univariate analysis, we did not find that nodule size was significantly associated with malignancy of PCTNs, and the finding was in accordance with previous studies (11, 19, 30). Hence, nodule size has not been described as a risk factor of malignant PCTNs. Nevertheless, size was considered as an important parameter to predict the malignancy in solid thyroid nodules. At present, many studies on nodule size are available, but no consensus as to its influence on probability of malignancy. Some studies have demonstrated that larger lesions had lower risk of malignancy (31, 32), while the other studies reached opposite conclusions (33, 34). The majority studies (31, 33) indicates that size threshold for malignant risk was approximately 2.0 cm in solid nodules, and cutoff value 2cm have been adopted in the study of PCTNs (12). Therefore, we divided the PCTNs into two groups to evaluate the impact of nodule size on the diagnostic efficacy of the scoring system. It was found that the effect of nodule size on the scoring system was nonsignificant, and the scoring system exhibited a high diagnostic accuracy in 2 groups of nodules with different sizes.

There are several limitations in our study. First, this study included some benign PCTNs for which only cytological results were available; for these lesions, pathological confirmation was absent or only a limited follow-up evaluation was performed. Second, we did not include all benign PCTNs. All nodules included in the study were removed by FNA or surgery due to suspicious ultrasound features or clinical indications. Therefore, we could not estimate the percentage of malignant PCTNs in our study, which may have been affected by selection bias to some extent. Third, this study did not investigate the inherent relationship between suspicious US features, like in the study of Zhang M et al. (22), but it may provide some new insights due to the more detailed classification of US features.

In conclusion, a scoring system based on the US features of PCTNs was developed to predict the risk of malignant PCTN in this study. This model provides a noninvasive, effective, and easy-to-use method that can be clinically implemented to identify malignant PCTNs preoperatively. This method is expected to assist radiologists in diagnosis and clinical decision making.

## Data Availability Statement

Due to the privacy of patients, the data related to patients cannot be available for public access but can be obtained from the corresponding author on reasonable request approved by the institutional review board of First Hospital of Shanxi Medical University.

## Ethics Statement

This retrospective study was approved by the Ethics Review Committee of First Hospital of Shanxi Medical University. The requirement for informed consent of patients was waived.

## Author Contributions

YX, LL, and JZ designed the study. YX, FL, YS, and XY collected the clinical data, ultrasonography images and histopathological/cytopathology results. FL and YX processed the clinical and images data. YS and YX performed the statistical analysis. YX and FL drafted and revised the manuscript. All authors contributed to the article and approved the submitted version.

## Funding

This work was supported by Application Basic Research Project of Science and Technology Department of Shanxi Province (201801D121340), and Key Research and Development Program of Science and Technology Department of Shanxi Province (201903D321190).

## Conflict of Interest

The authors declare that the research was conducted in the absence of any commercial or financial relationships that could be construed as a potential conflict of interest.

## Publisher’s Note

All claims expressed in this article are solely those of the authors and do not necessarily represent those of their affiliated organizations, or those of the publisher, the editors and the reviewers. Any product that may be evaluated in this article, or claim that may be made by its manufacturer, is not guaranteed or endorsed by the publisher.
