# Supplementary material for: A Scoring System for Assessing the Risk of Malignant Partially Cystic Thyroid Nodules Based on Ultrasound Features
Source: Front Oncol. 2021 Oct 6;11:731779. doi: 10.3389/fonc.2021.731779 (PMC8526936; doi:10.3389/fonc.2021.731779)
Supplement: Supplementary file 1 [file DataSheet_1.pdf]

Logistic Regression

Numeric Results

Power	N	Pcnt N X=1	P0	P1	Odds Ratio	R Squared	Alpha	Beta
0.79833	194	0.380	0.050	0.920	218.500	0.360	0.05000	0.20167
0.89949	280	0.380	0.050	0.920	218.500	0.360	0.05000	0.10051

References

Hsieh, F.Y., Block, D.A., and Larsen, M.D. 1998. 'A Simple Method of Sample Size Calculation for Linear and Logistic Regression', Statistics in Medicine, Volume 17, pages 1623-1634.

Report Definitions

Power is the probability of rejecting a false null hypothesis. It should be close to one.
N is the size of the sample drawn from the population.
P0 is the response probability at the mean of X.
P1 is the response probability when X is increased to one standard deviation above the mean.
Odds Ratio is the odds ratio when P1 is on top. That is, it is [P1/(1-P1)]/[P0/(1-P0)].
R-Squared is the R2 achieved when X is regressed on the other independent variables in the regression.
Alpha is the probability of rejecting a true null hypothesis.
Beta is the probability of accepting a false null hypothesis.

Summary Statements

A logistic regression of a binary response variable (Y) on a binary independent variable (X) with a sample size of 194 observations (of which 100% are in the group X=0 and 0% are in the group X=1) achieves 80% power at a 0.05000 significance level to detect a change in Prob(Y=1) from the baseline value of 0.050 to 0.920. This change corresponds to an odds ratio of 218.500. An adjustment was made since a multiple regression of the independent variable of interest on the other independent variables in the logistic regression obtained an R-Squared of 0.360.

Dropout-Inflated Sample Size

Dropout Rate	Sample Size N	Dropout-Inflated Enrollment Sample Size N'	Expected Number of Dropouts D
20%	194	243	49
20%	280	350	70

Definitions

Dropout Rate (DR) is the percentage of subjects (or items) that are expected to be lost at random during the course of the study and for whom no response data will be collected (i.e. will be treated as "missing").
N is the evaluable sample size at which power is computed. If N subjects are evaluated out of the N' subjects that are enrolled in the study, the design will achieve the stated power.
N' is the total number of subjects that should be enrolled in the study in order to end up with N evaluable subjects, based on the assumed dropout rate. After solving for N, N' is calculated by inflating N using the formula N' = N / (1 - DR), with N' always rounded up. (See Julious, S.A. (2010) pages 52-53, or Chow, S.C., Shao, J., and Wang, H. (2008) pages 39-40.)
D is the expected number of dropouts. D = N' - N.

Logistic Regression

Chart Section

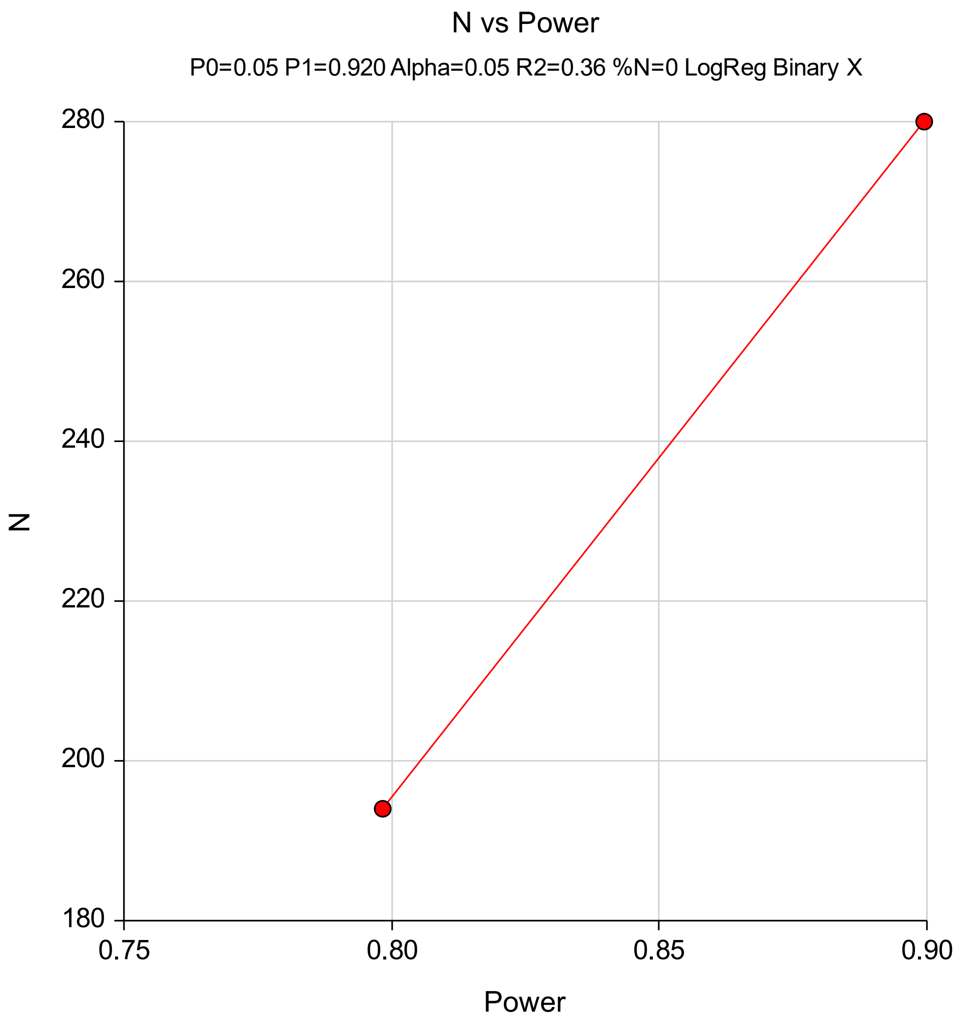

Logistic Regression

Procedure Input Settings

Autosaved Template File
d:\Documents\PASS 15\Procedure Templates\Autosave\Logistic Regression - Autosaved 2021_5_18-13_21_59.t123

Design Tab
Solve For: Sample Size
Alternative Hypothesis: One-Sided
Power: 0.8 0.90
Alpha: 0.05
P0 (Baseline Probability that Y=1): 0.05
Use P1 or Odds Ratio: P1
P1 (Alternative Probability that Y=1):
0.92
R-Squared of X1 with Other X's: 0.36
X1 (Independent Variable of Interest):
Binary (X = 0 or 1)
Percent of N with X1=1: 0.38
