# Supplementary material for: A Scoring System for Assessing the Risk of Malignant Partially Cystic Thyroid Nodules Based on Ultrasound Features
Source: Front Oncol. 2021 Oct 6;11:731779. doi: 10.3389/fonc.2021.731779 (PMC8526936; doi:10.3389/fonc.2021.731779)
Supplement: Supplementary file 2 [file DataSheet_2.pdf]

Tests for One ROC Curve

Numeric Results for Testing AUC0 = AUC1 with Discrete (Rating) Data

Test Type = One-Sided. FPR1 = 0.01. FPR2 = 0.10. B = 0.01.

Target Power	Actual Power	N+	N-	N	Target R	Actual R	AUC0'	AUC1'	Diff'	AUC0	AUC1	Diff	Alpha
0.80	0.80662	48	18	66	0.38	0.38	0.8700	0.8000	-0.0700	0.0679	0.0560	-0.0119	0.050
0.90	0.90175	67	25	92	0.38	0.37	0.8700	0.8000	-0.0700	0.0679	0.0560	-0.0119	0.050

References

Hanley, J. A. and McNeil, B. J. 1983. 'A Method of Comparing the Areas under Receiver Operating Characteristic Curves Derived from the Same Cases.' Radiology, 148, 839-843. September, 1983.

Obuchowski, N. and McClish, D. 1997. 'Sample Size Determination for Diagnostic Accuracy Studies Involving Binormal ROC Curve Indices.' Statistics in Medicine, 16, pages 1529-1542.

Report Definitions

Target Power is the desired power value (or values) entered in the procedure. Power is the probability of rejecting a false null hypothesis.

Actual Power is the power obtained in this scenario. Because N+ and N- are discrete, this value is often (slightly) larger than the target power.

N+ and N- are the number of items sampled from each population.

N is the total sample size, N+ + N-.

Target R is the desired ratio (or ratios) of R entered in the procedure. R is the ratio of N- to N+, so that N- = R × N+.

Actual R is the value for R obtained in this scenario. Because N+ and N- are discrete, this value is sometimes slightly different than the target R.

AUC0' and AUC1' are the adjusted areas under the ROC curve for the null and alternative hypotheses, respectively.

Diff' is AUC1 - AUC0. This is the adjusted difference to be detected.

AUC0 and AUC1 are the actual areas under the ROC curve for the null and alternative hypotheses, respectively.

Diff is AUC1 - AUC0. This is the difference to be detected.

Alpha is the probability of rejecting a true null hypothesis.

FPR1, FPR2 are the lower and upper bounds on the false positive rates.

B is the ratio of the standard deviations of the negative and positive groups.

Summary Statements

A sample of 48 from the positive group and 18 from the negative group achieves 81% power to detect a difference of 0.0700 between the area under the ROC curve (AUC) under the null hypothesis of 0.8700 and an AUC under the alternative hypothesis of 0.8000 using a one-sided z-test at a significance level of 0.050. The data are discrete (rating scale) responses. The AUC is computed between false positive rates of 0.01 and 0.10. The ratio of the standard deviation of the responses in the negative group to the standard deviation of the responses in the positive group is 0.01.

Dropout-Inflated Sample Size

	Sample Size			Dropout-Inflated Enrollment Sample Size			Expected Number of Dropouts		
Dropout Rate	N+	N-	N	N+'	N-'	N'	D+	D-	D
20%	48	18	66	60	23	83	12	5	17
20%	67	25	92	84	32	116	17	7	24

Tests for One ROC Curve

Definitions

Dropout Rate (DR) is the percentage of subjects (or items) that are expected to be lost at random during the course of the study and for whom no response data will be collected (i.e. will be treated as "missing").

N+, N-, and N are the evaluable sample sizes at which power is computed. If N+ and N- subjects are evaluated out of the N+' and N-' subjects that are enrolled in the study, the design will achieve the stated power.

N+', N-', and N' are the number of subjects that should be enrolled in the study in order to end up with N+, N-, and N evaluable subjects, based on the assumed dropout rate. After solving for N+ and N-, N+' and N-' are calculated by inflating N+ and N- using the formulas N+' = N+ / (1 - DR) and N-' = N- / (1 - DR), with N+' and N-' always rounded up. (See Julious, S.A. (2010) pages 52-53, or Chow, S.C., Shao, J., and Wang, H. (2008) pages 39-40.)

D+, D-, and D are the expected number of dropouts. D+ = N+' - N+, D- = N-' - N-, and D = D+ + D-.

Chart Section

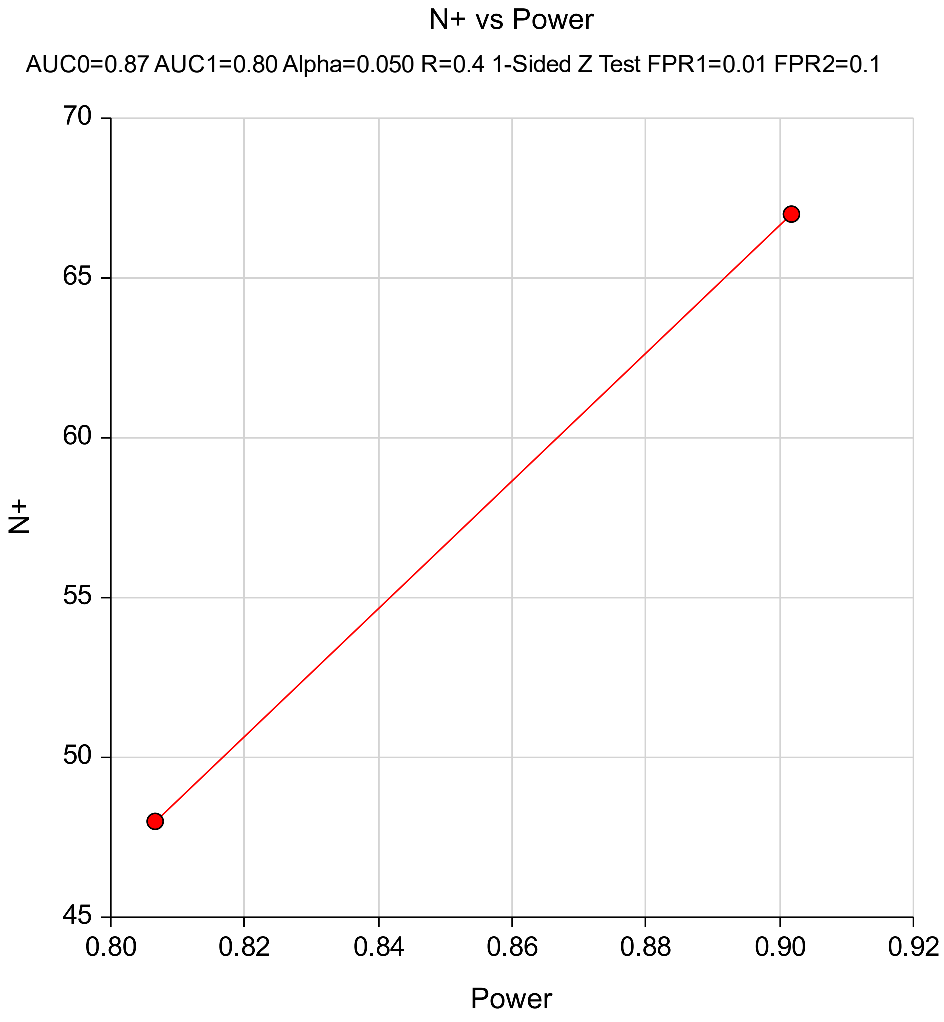

Tests for One ROC Curve

Procedure Input Settings

Autosaved Template File
d:\Documents\PASS 15\Procedure Templates\Autosave\Tests for One ROC Curve - Autosaved 2021_5_18-13_16_0.t158

Design Tab

Solve For:	Sample Size
Alternative Hypothesis:	One-Sided Test
Power:	0.8 0.90
Alpha:	0.05
Group Allocation:	Enter R = N-/N+, solve for N+ and N-
R:	0.38
AUC0 (Area Under Curve|H0):	0.87
AUC1 (Area Under Curve|H1):	0.8
Lower FPR:	0.01
Upper FPR:	0.10
Type of Data:	Discrete (Ratings)
B (SD Ratio = SD-/SD+):	0.01
