# Supplementary material for: A Scoring System for Assessing the Risk of Malignant Partially Cystic Thyroid Nodules Based on Ultrasound Features
Source: Front Oncol. 2021 Oct 6;11:731779. doi: 10.3389/fonc.2021.731779 (PMC8526936; doi:10.3389/fonc.2021.731779)
Supplement: Supplementary file 3 [file DataSheet_3.docx]

Supplementary Material

# Supplementary Figures

**Supplementary Figure 1.** Calibration curve of the logistics regression model in primary cohort. The resultant curve and ideal curve had good consistency, which was confirmed by Hosmer-Lemeshow test.


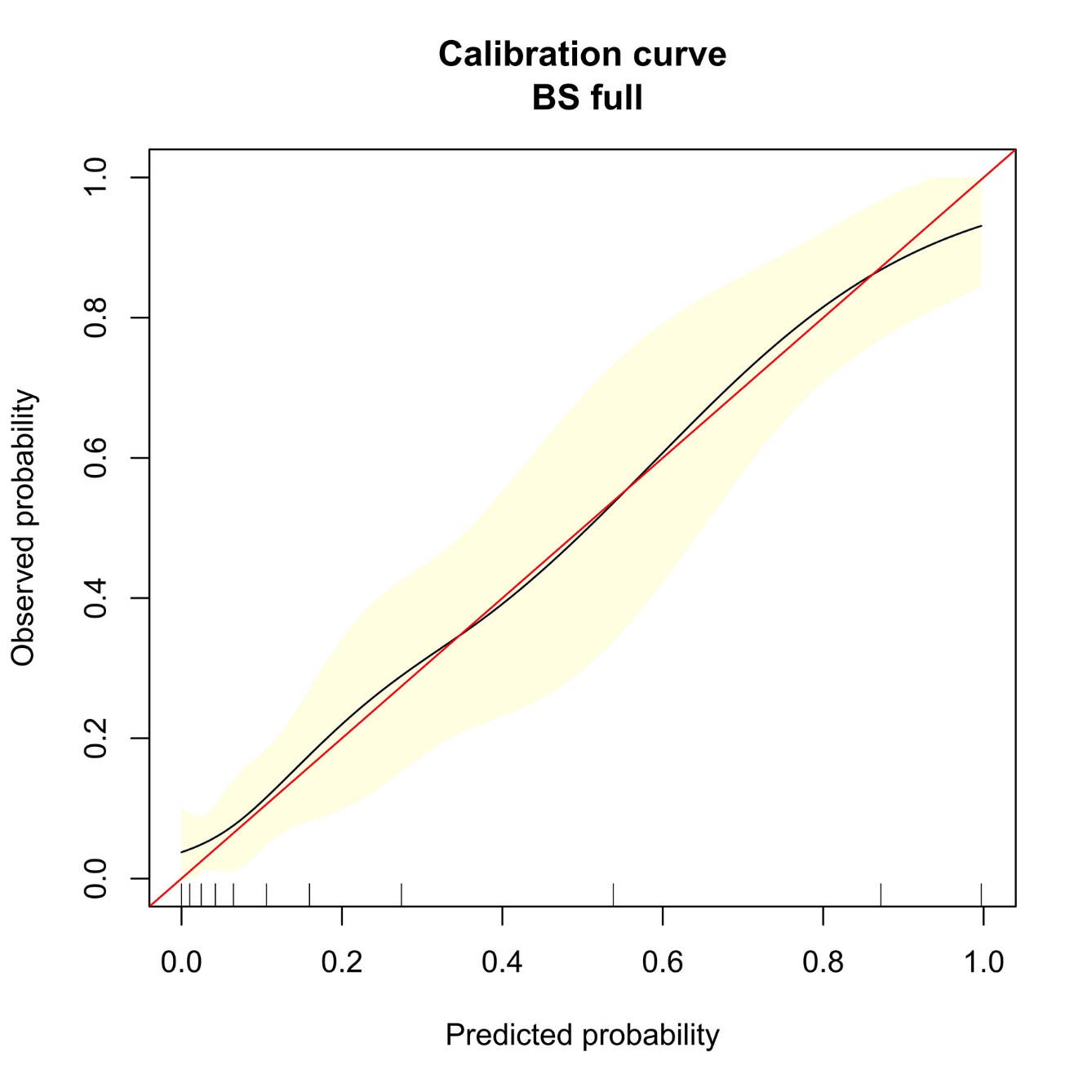


**Supplementary Figure 2.** Confusion matrix of the scoring system in the primary cohort (A) and external validation cohort (B), which was to show the diagnostic ability of the scoring system.

**Figure 2A** **2B**


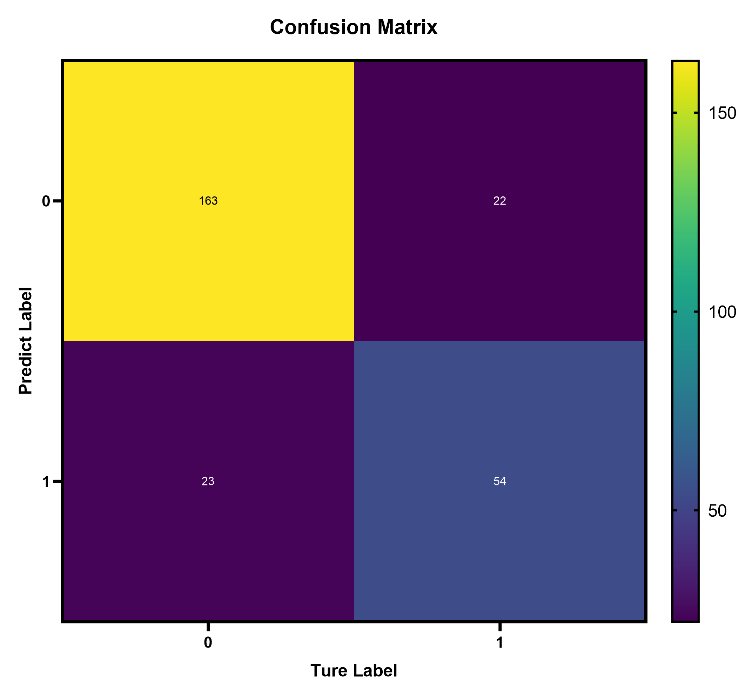

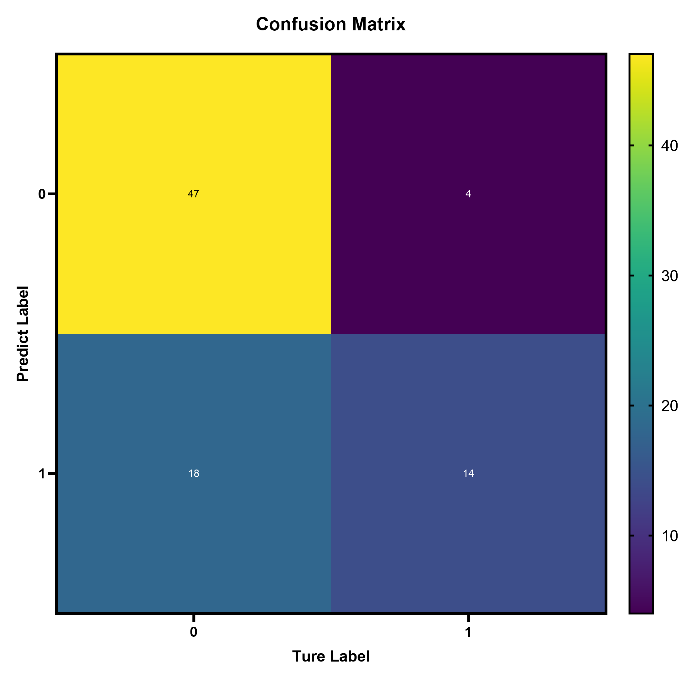


**Supplementary Tables**

**Supplementary Table 1.**

**Table 1:** The sensitivity and specificity for all scores of primary cohort and validation cohort.

|  | Primary cohort | | Validation cohort | |
| --- | --- | --- | --- | --- |
|  | Specificity | Sensitivity | Specificity | Sensitivity |
| ≥1 | 0.00 (0.0 - 2.0) | 100.00 (95.3 - 100.0) | 0.00 (0.0 - 5.5） | 100.00 (81.5 -100.0） |
| ＞1 | 13.98 (9.3 - 19.8) | 98.68 (92.9 - 100.0) | 7.69 (2.5 - 17.0) | 100.00 (81.5 -100.0） |
| ＞2 | 43.55 (36.3 - 51.0) | 97.37 (90.8 - 99.7) | 32.31 (21.2 - 45.1) | 100.00 (81.5 -100.0） |
| ＞3 | 72.04 (65.0 - 78.4) | 82.89 (72.5 - 90.6) | 53.85 (41.0 - 66.3) | 88.89 (65.3 - 98.6） |
| ＞4 | 87.63 (82.0 - 92.0) | 71.05 (59.5 - 80.9) | 72.31 (59.8 - 82.7) | 77.78 (52.4 - 93.6） |
| ＞5 | 95.16 (91.0 - 97.8) | 56.58 (44.7 - 67.9) | 90.77 (81.0 - 96.5) | 50.00 (26.0 - 74.0） |
| ＞6 | 98.39 (95.4 - 99.7) | 44.74 (33.3 - 56.6) | 98.46 (91.7 - 100.0) | 27.78 (9.7 - 53.5） |
| ＞7 | 100.00 (98.0 - 100.0) | 26.32 (16.9 - 37.7) | 98.46 (91.7 - 100.0) | 5.56 (0.1 - 27.3） |
| ＞8 | 100.00 (98.0 - 100.0) | 11.84 (5.6 - 21.3) | 100.00 (94.5 - 100.0) | 0.00 (0.0 - 18.5） |
| ＞9 | 100.00 (98.0 - 100.0) | 5.26 (1.5 - 12.9) | - | - |
| ＞10 | 100.00 (98.0 - 100.0) | 1.32 (0.03 - 7.1) | - | - |
| ＞11 | 100.00 (98.0 - 100.0) | 0.00 (0.0 - 4.7) | - | - |
